# Pachydermodactyly: A benign mimicker of inflammatory arthritis in a young man

**DOI:** 10.1016/j.jdcr.2026.05.014

**Published:** 2026-05-12

**Authors:** Tadele Demilew Chekol, Bekele Taye Feleke

**Affiliations:** Assistant Professor of Internal Medicine, Debre Markos University, Debre Markos, Ethiopia

**Keywords:** digital fibromatosis, inflammatory arthritis mimic, pachydermodactyly, periarticular swelling, proximal interphalangeal joints

## Introduction

Pachydermodactyly (PDD) is a rare, benign digital fibromatosis characterized by asymptomatic periarticular soft tissue swelling, most commonly affecting the proximal interphalangeal (PIP) joints. It primarily occurs in adolescent and young adult males and is frequently associated with repetitive mechanical trauma. Due to its resemblance to inflammatory arthropathies, PDD is often misdiagnosed, leading to unnecessary investigations and treatment.

We report a case of PDD in a young man initially suspected to have inflammatory arthritis and highlight key features for differentiation.

## Case report

A 20-year-old male presented with a 6-year history of progressive swelling of all fingers except the thumbs. The swelling was painless and not associated with stiffness, erythema, or functional limitation. He reported a longstanding habit of rubbing and twisting his fingers since childhood.

He had previously received 2 local corticosteroid injections without clinical improvement due to suspected inflammatory arthritis.

There was no history of systemic symptoms, trauma, or family history of rheumatologic disease.

### Examination

**Fig 1:**
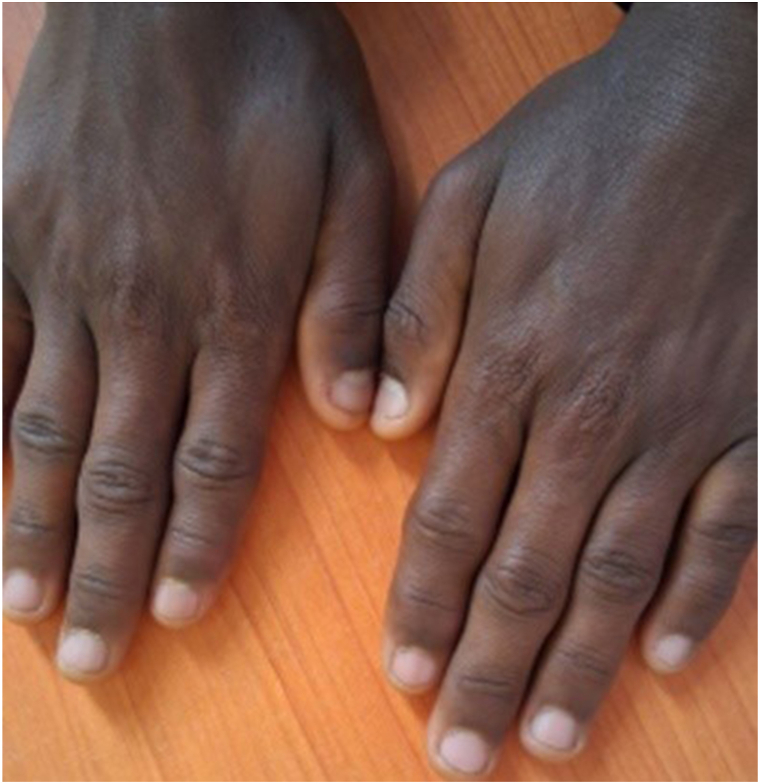
Symmetrical, nontender soft tissue swelling of the proximal interphalangeal joints of the index, middle, ring, and small fingers with sparing of the thumbs.

Examination revealed bilateral, symmetrical, nontender soft tissue swelling over the lateral aspects of the PIP joints, sparing the thumbs (Fig 1). Skin overlying the joints was normal without erythema or warmth. Range of motion was fully preserved. There was no joint tenderness or synovitis.

### Investigations

**Fig 2:**
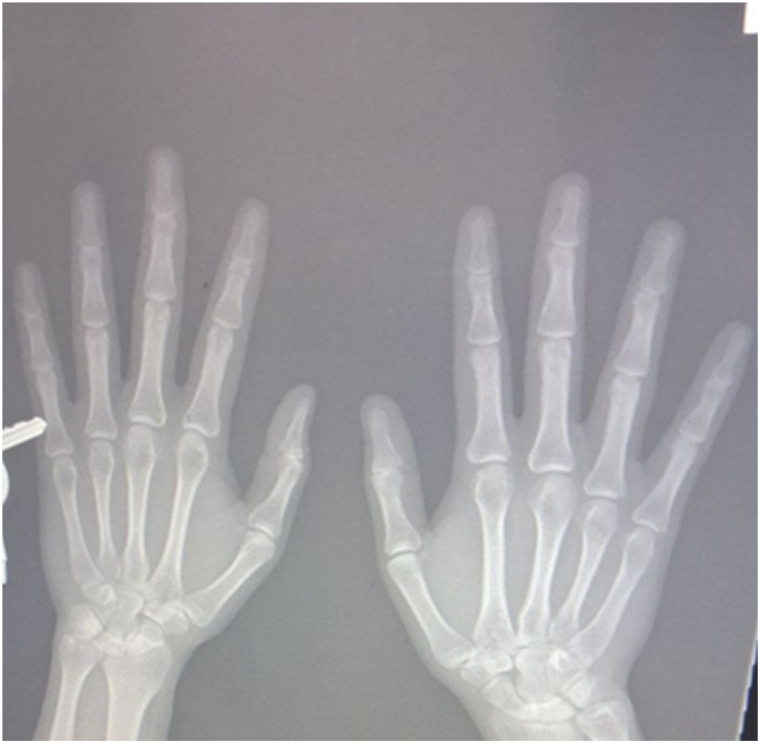
Normal hand radiograph showing no erosions, joint space narrowing, or bony abnormalities.

• Complete blood count: normal
• Erythrocyte sedimentation rate and C-reactive protein: normal
• Rheumatoid factor: negative
• Antinuclear antibody: negative
• Hand radiographs (Fig 2): normal, no erosions, or joint space narrowing

• Ultrasound: mild periarticular soft tissue thickening without synovitis or joint involvement

### Diagnosis and management

Based on characteristic clinical findings, normal inflammatory markers, and absence of joint involvement, a diagnosis of pachydermodactyly was made.

The patient was reassured regarding the benign nature of the condition and advised to avoid repetitive mechanical trauma. Psychiatric evaluation was recommended to assess possible compulsive behavior. No pharmacologic therapy was initiated.

## Literature review

### Epidemiology and etiology

PDD is uncommon, with a strong male predominance and onset typically during adolescence or early adulthood. Repetitive mechanical trauma (habitual rubbing, interlocking, or knuckle manipulation) is considered a major contributing factor.^1^

### Pathogenesis

PDD represents localized fibromatosis of periarticular soft tissue rather than an inflammatory process. Histopathology, when performed, shows dermal collagen thickening and fibroblast proliferation but is not required for diagnosis.^2^

### Clinical features

Typical features include:

• Painless, slowly progressive swelling of PIP joints
• Symmetrical involvement (usually second–fourth digits)
• Sparing of thumbs
• Absence of pain, stiffness, or systemic symptoms
• Normal laboratory findings
• No radiographic joint destruction is seen (Fig 2). Laboratory and imaging studies are typically normal and primarily serve to exclude alternative diagnoses.^3^^,^^4^

### Differential diagnosis

The main diagnostic challenge lies in distinguishing PDD from inflammatory arthropathies^5^ (Table I).

**Table I:** Differential diagnosis of PIP joint swelling

Feature	PDD	Rheumatoid arthritis	Psoriatic Arthritis
Pain	Absent	Present	Present
Morning stiffness	Absent	Present	Present
Swelling type	Soft tissue	Synovial	Synovial
Inflammation signs	Absent	Present	Present
Systemic features	Absent	Present	Possible
Labs	Normal	RF/anti-CCP +	Variable
Joint damage on imaging	Absent	Present	Possible
Skin/nail findings	Absent	Absent	Present

Other differential diagnoses include juvenile idiopathic arthritis and juvenile hyaline fibromatosis, the latter distinguished by systemic involvement such as gingival hypertrophy, skin lesions, and bone changes.^6^

*PDD*, Pachydermodactyly; *PIP*, proximal interphalangeal; *RF*, rheumatoid factor.

### Management

Management is conservative and includes:

• Reassurance and education
• Behavioral modification to reduce repetitive trauma
• Psychiatric assessment in selected cases
• Avoidance of unnecessary immunosuppressive therapy

## Discussion

Pachydermodactyly is a benign condition that can closely mimic inflammatory arthritis and lead to misdiagnosis and unnecessary treatment, as demonstrated in this case where the patient received corticosteroid injections without benefit.^7^ Recognition of PDD is critical to avoid iatrogenic interventions, including unnecessary corticosteroid therapy. Key distinguishing features include:

• Painless periarticular PIP swelling
• Symmetrical involvement of fingers
• Normal inflammatory markers (erythrocyte sedimentation rate, C-reactive protein, rheumatoid factor, and antinuclear antibody)
• Absence of joint space narrowing or erosions
• History of repetitive mechanical behavior

### Clinical importance

• Prevents unnecessary immunosuppressive therapy
• Avoids invasive investigations
• Reduces patient anxiety and healthcare burden

#### Key points

• Consider PDD in young males with painless PIP swelling
• Normal labs and imaging help exclude inflammatory arthritis
• Behavioral history is a crucial diagnostic clue
• Management is reassurance and habit modification

## Conclusion

PDD is a rare but important benign cause of periarticular finger swelling that can closely mimic inflammatory arthritis. Awareness of its characteristic clinical presentation and normal laboratory findings is essential to avoid misdiagnosis and unnecessary treatment.

## Conflicts of interest

None disclosed.

## References

[1] Beltraminelli H., Itin P. (2009). Pachydermodactyly--just a sign of emotional distress. Eur J Dermatol.

[2] Alrubaiaan M.T., Alharthi Y.H., Alfaraj S. (2025). Pachydermodactyly: an underdiagnosed condition in adolescence-a case report and literature review. Case Rep Dermatol Med.

[3] Hussain S., Ehtesham M., Almas T., Aldei A. (2021). Painful pachydermodactyly in a 39-year-old woman: a case report and review of the literature. Ann Med Surg (Lond).

[4] Vázquez Fernández R., Maneiro Fernández J.R., Cervantes Pérez E.C., Mera Varela A. (2021). Pachydermodactyly: a systematic review. Ir J Med Sci.

[5] El-hallak M., Lovell D. (2013). Pachydermodactyly mimicking juvenile idiopathic arthritis. Arthritis Rheum.

[6] Fathalla B.M., Goldsmith D.P. (2009). Pachydermatodactyly mimics polyarticular juvenile idiopathic arthritis. J Pediatr.

[7] Rukavina I., Frković M., Sestan M. (2025). Pachydermodactily - the great imitator of arthritis: a case series. Croat Med J.

